# Targeted RNA Degradation as a Promising Therapeutic Strategy

**DOI:** 10.3390/ijms262110767

**Published:** 2025-11-05

**Authors:** Sivakumar Komachankandy, Yeongju Lee

**Affiliations:** 1 Department of Chemistry, Pusan National University, Busan 46241, Republic of Korea; isivakumarkk@gmail.com; 2 Institute for Future Earth, Pusan National University, Busan 46241, Republic of Korea

**Keywords:** targeted RNA degradation, RIBOTAC, bleomycin, PINAD, small-molecule RNA degrader

## Abstract

RNAs have recently emerged as versatile therapeutic targets, broadening the scope of drug discovery beyond the conventional protein-centered paradigm. Small-molecule-induced RNA degradation has been established as a promising approach, with novel modalities such as Ribonuclease-Targeting Chimeras (RIBOTACs), bleomycin-conjugated degraders, and imidazole-based RNA degrader demonstrating strong potential. These strategies selectively eliminate disease-associated RNAs by harnessing endogenous ribonucleases, redirecting the nucleic acid-cleaving activity of natural products, or incorporating catalytic warheads. Recent studies have validated therapeutic applications across cancer, neurodegenerative disorders, and viral infections, underscoring the wide-ranging impact of this strategy. Nevertheless, key challenges remain, including the development of more potent recruiters, diversification of degradation mechanisms, optimization of linker chemistry, and overcoming pharmacokinetic limitations. With continued innovation, RNA degraders are expected to evolve into a robust therapeutic platform that expands the druggable space and enables new treatment opportunities for diseases once considered untreatable.

## 1. Introduction

### 1.1. Targeting RNAs as Promising Therapeutic Strategy

RNA transcripts are now recognized as critical regulators of diverse biological processes. Historically, RNA was considered a passive messenger, transmitting genetic information from DNA to guide protein synthesis. However, advances in molecular biology have revealed that RNA plays far broader roles, including direct regulation of gene expression and cellular functions through multiple mechanisms such as transcription [1], translation [2], splicing [3], RNA–protein interactions, and broader gene-regulatory networks [4]. Notably, although approximately 85% of the human genome is transcribed into RNA, only ~3% of these transcripts encode proteins, underscoring the predominance of noncoding RNAs (ncRNAs) [5]. Many ncRNAs are implicated in the pathogenesis of diseases, including cancer [6]. In contrast, drug discovery efforts have been predominantly focused on protein targets, with only ~15% of the ~20,000 human proteins currently considered druggable [7]. As a result, protein-targeted therapies address only a narrow spectrum of disease-related biomolecules, whereas RNA offers a substantially broader and underexplored therapeutic opportunity.

Among ncRNAs, microRNAs (miRNAs) are particularly well characterized. These ~22 nucleotide-long RNAs act post-transcriptionally by repressing or degrading target mRNAs. Dysregulation of miRNAs has been consistently associated with disease: for example, miR-21 is frequently upregulated in cancers and promotes cell proliferation and metastasis [8], miR-155 is aberrantly elevated in immune and inflammatory disorders [9], and loss of tumor-suppressive miRNAs such as miR-34a contributes to defective apoptosis and enhanced tumor survival [10]. These examples demonstrate the broad involvement of miRNAs in oncogenesis, cardiovascular disease, and immune dysfunction.

Beyond miRNAs, other ncRNAs have also emerged as key regulators of pathology. Long noncoding RNAs (lncRNAs) such as MALAT1 and HOTAIR have been implicated in cancer progression and metastasis [11], while NEAT1 and XIST are linked to neurological disorders and tumor microenvironmental regulation [12,13]. Importantly, messenger RNAs (mRNAs) themselves can also serve as direct therapeutic targets. By targeting transcripts that encode proteins traditionally deemed “undruggable” due to the absence of suitable binding pockets, RNA-directed strategies enable the indirect suppression of disease-driving proteins.

Taken together, these insights highlight RNA as a promising and attractive therapeutic target. Targeting RNA including miRNAs, lncRNAs, and mRNAs offers opportunities to address disease mechanisms beyond the reach of protein-directed drugs, representing a significant broadening of therapeutic targets in the field of drug discovery.

### 1.2. Therapeutic Strategies to Target RNA with Oligonucleotides

RNA therapeutics were first introduced as innovative strategies to treat untreatable diseases with antisense oligonucleotides (ASOs), representing one of the earliest modalities. ASOs exploit the principle of RNA–RNA base pairing, discovered in 1956, to selectively recognize complementary sequences [14]. In 1998, the first ASO-based drug, fomivirsen, was approved by the FDA for the treatment of cytomegalovirus retinitis [15]. ASOs act mainly through two mechanisms: (i) promoting targeted RNA degradation, or (ii) modulating pre-mRNA splicing. In the degradation pathway, ASO hybridization leads to the formation of a DNA–RNA duplex, which is recognized and cleaved by RNase H, thereby eliminating the target RNA. In the splicing modulation pathway, ASOs sterically block splicing factors from accessing pre-mRNA regulatory sequences, thereby correcting aberrant splicing events associated with disease.

Small interfering RNAs (siRNAs) further expanded the RNA therapeutic landscape through the RNA interference (RNAi) pathway. Once delivered into cells, the siRNA duplex unwinds, and the guide strand associates with Argonaute proteins to form the RNA-induced silencing complex (RISC). This complex recognizes target mRNAs with sequence specificity and silences their expression. Several siRNA-based drugs have been approved to date, including patisiran, givosiran, lumasiran, inclisiran, vutrisiran, and nedosiran [16,17,18]. These approvals underscore the clinical maturation of RNAi-based therapies.

Peptide nucleic acids (PNAs) represent another synthetic platform for RNA-targeted therapeutics. PNAs are DNA mimics in which the sugar–phosphate backbone is replaced by N-(2-aminoethyl) glycine units, providing exceptional thermal, pH, and ionic stability, as well as resistance to nucleases and proteases. PNAs bind with high affinity, capable of invading DNA duplexes or binding complementary RNA. However, early PNAs displayed poor solubility and aggregation, which limited their therapeutic utility. These drawbacks were addressed in 2006 by introducing γ-modified PNAs with a hydroxymethyl group, which promoted a preorganized helical structure and improved solubility [19]. Consequently, PNAs are now explored as versatile agents capable of silencing disease-associated genes or targeting structured RNAs.

### 1.3. Therapeutic Strategies to Target RNA with Small Molecules

Beyond synthetic oligonucleotides, the structural features of RNA itself have become a major focus for drug discovery. While nucleic acids typically follow Watson–Crick base-pairing (A–U and G–C), RNAs can fold into complex secondary and tertiary structures, including hairpins, internal loops, pseudoknots, triplexes, G-quadruplexes, and bulge. These structural motifs form stable, well-defined binding pockets that are highly amenable to small-molecule recognition. For example, small molecules binding to riboswitches in 5′ UTRs can induce conformational changes that repress gene expression [20]. Others have been shown to interact with structural elements in Drosha or Dicer processing sites, thereby preventing proper miRNA maturation [21]. Historically, bacterial ribosomes were the first RNA targets of small molecules, exemplified by streptomycin, which binds the 16S rRNA decoding site [22]. In addition, viral RNAs often rely on conserved internal ribosomal entry sites (IRES) for translation, and small molecules that disrupt IRES-mediated ribosome recruitment have been identified in pathogens such as HCV and enteroviruses [23,24].

As RNA structural complexity becomes partially well characterized, small-molecule targeting of RNA has emerged as an attractive therapeutic strategy. This is largely driven by the favorable pharmacological properties of small molecules, including cell permeability, metabolic stability, and oral bioavailability, making them highly appealing compared to larger oligonucleotide therapeutics.

Inspired by the remarkable structural diversity of RNA, which gives rise to a wide array of unique recognition motifs for small molecules, the two-dimensional combinatorial screening (2DCS) platform was developed [25]. This powerful approach systematically measures the binding preferences of diverse chemical scaffolds against large libraries of structured RNAs that capture recurrent secondary and tertiary motifs, including hairpins, bulges, internal loops, pseudoknots, and G-quadruplex elements. By interrogating thousands of RNA–ligand interactions in parallel, 2DCS establishes privileged chemotypes for distinct RNA folds and generates a binding landscape that can be used to rationally prioritize lead structures. Representative successes of 2DCS include the discovery of ligands that bind to the oncogenic pri-miR-210 hairpin [26], thereby inhibiting its maturation. These findings highlight the ability of 2DCS to uncover small molecules that engage RNA structures previously considered intractable.

The data generated by 2DCS has been systematized into a searchable informatics framework known as INFORNA [27], developed by the Disney group. INFORNA integrates RNA secondary-structure predictions across the human transcriptome with 2DCS-derived rules of motif–ligand recognition, thus enabling the rational design of selective RNA binders directly from sequence information. By applying this strategy, researchers have successfully identified numerous inhibitors of disease-relevant RNAs. For instance, INFORNA-guided discovery yielded small molecules that block the processing of the oncogenic miR-21, thereby reducing the levels of its mature form [28]. In addition, the same platform enabled the design of **TGP-377**, a compound that targets precursor microRNA-377 (pre-miR-377) and enhances VEGFA expression [29]. Together, these advances highlight how INFORNA has transformed small-molecule RNA targeting from serendipitous discovery into a predictable and generalizable design process.

**Figure 1:**
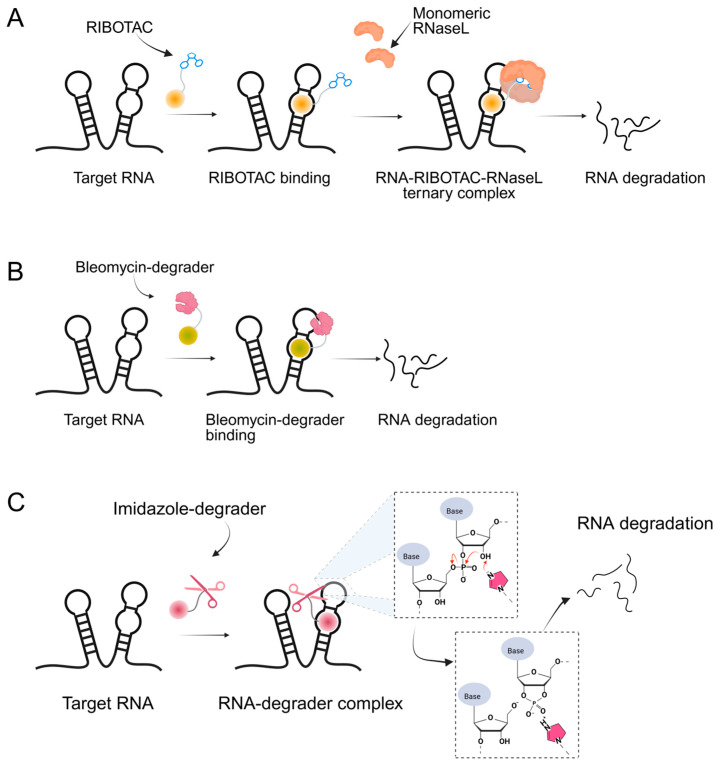
Graphical representation of mechanism of targeted RNA degradation by (**A**) RIBOTACs, (**B**) Bleomycin-conjugated degraders and (**C**) Imidazole-conjugated degraders.

Building upon these ligand-discovery methods, new approaches have been developed to expand the scope from occupancy-based inhibition to active degradation of RNA. The most prominent is Ribonuclease-Targeting Chimeras (RIBOTACs), which harness a natural cellular mechanism by recruiting the endogenous human RNase L to the target RNA [30]. In RIBOTACs, a validated RNA-binding small molecule is tethered to an RNase-L-recruiting module, so that proximity-induced dimerization and activation of RNase L cleave the bound RNA in cells, without introducing any exogenous nuclease [28,31,32] (Figure 1A). Although these synthetic recruiters generally require higher concentrations than native 2′-5′-oligoadenylates, they provide drug-like handles for selective degradation when coupled to high-affinity RNA binders.

In parallel, bleomycin conjugates have been engineered to redirect the intrinsic nucleic acid-cleaving activity of this natural product toward specific RNA targets (Figure 1B), exemplified by Cugamycin [33], which selectively degrades toxic r(CUG) repeats in myotonic dystrophy models. Similarly, synthetic direct RNA-cleaving warheads, such as imidazole-based catalysts or related chemicals [32,34,35], have been incorporated into chimeric molecules to enable sequence- or structure-dependent RNA scission (Figure 1C). Together, these complementary approaches broaden the toolbox for targeted RNA degradation beyond RNase L recruitment. Herein, we focus on the emerging field of small-molecule-based targeted RNA degradation.

## 2. RIBOTAC

### 2.1. RIBOTACs Targeting microRNAs

The Disney group established the first small-molecule RIBOTAC by conjugating an RNA-binding ligand for the primary microRNA-96 (pri-miR-96) to a short 2′-5′-oligoadenylate (2′-5′A_4_) that recruits and locally activates endogenous RNase L [36] (Table 1). By utilizing the INFORNA platform, the authors identified a potent pre-miR-96 binder, **1a**, and converted it into pri-miR-96-targeting RIBOTAC. The designed pri-miR-96 RIBOTAC, **2**, induces site-selective cleavage of pri-miR-96 in vitro and in cells at 200 nM concentration. Linker optimization identified the shortest spacer as optimal, preserving high affinity for the Drosha hairpin site and inducing RNase L-mediated cleavage of the native pri-miR-96. In contrast, pri-miR-96 RIBOTACs with longer spacers still induced RNA cleavage but with reduced efficacy. In triple-negative breast cancer cells, compound **2** reduced both pri- and mature miR-96 (mat-miR-96), formed a ternary complex with RNase L on-target. In addition, degradation of miR-96 with **2** derepressed FOXO1 and triggered apoptosis in cancer, but not in normal breast cells. Ribonuclease recruitment improved more than 5-fold potency versus the parent binder. It indicates that the RIBOTAC-mediated RNA cleavage was catalytic and sub-stoichiometric (~3.1 pri-miR-96 per molecule of chimera), highlighting that programmable nuclease recruitment by small molecules can drive selective RNA decay and providing a new therapeutic method targeting RNAs.

Whereas compound **2** employed 2′-5′A_4_ as an RNase L recruiter, the targeting of miR-21 was achieved using a fully small-molecule recruiter, **C1** [42]. This represented the first example in which both the RNA ligand and the RNase L recruiter were entirely composed of small molecules [28]. Using the INFORNA design platform, the authors identified a fragment binder to the Dicer site of pre-miR-21, optimized it into a dimeric ligand with improved affinity and selectivity. The dimeric ligand was subsequently conjugated to **C1**. The resulting **pre-miR-21 RIBOTAC** efficiently recruited RNase L to induce catalytic degradation of pre-miR-21 in cancer cells, exhibiting nanomolar potency and enhanced efficacy compared with its parent binder. In addition, the compound reduced mat-miR-21, restored levels of tumor suppressors such as PDCD4 and PTEN, and inhibited invasion and metastasis of breast, lung, and melanoma cancer cell lines. Furthermore, RT-qPCR profiling of miRNAs in MDA-MB-231 cells treated with the pre-miR-21 RIBOTAC revealed a pronounced reduction in miR-21 levels with minimal changes across other miRNAs, confirming its high selectivity. In parallel, no significant off-target effects were observed on abundant RNA species, including rRNAs, tRNAs, and mRNAs. Notably, in a mouse xenograft model of metastatic breast cancer, **pre-miR-21 RIBOTAC** suppressed lung metastasis by triple-negative breast cancer cells, reduced metastatic nodules, and increased expression of PDCD4 protein in tumor tissue.

Zhang et al. explored a drug repositioning strategy by converting the clinically used receptor tyrosine kinase (RTK) inhibitor dovitinib [43] into an RNA-targeted RIBOTAC [37]. While dovitinib alone reduced mat-miR-21 levels by about 30% at 5 μM, the **dovitinib-RIBOTAC** achieved the same effect at 0.2 μM, representing more than a 25-fold increase in potency. In addition to improved potency, the conjugated degrader also exhibited enhanced specificity, effectively reversing miR-21-associated phenotypes across multiple cancer cell lines. Transcriptome-wide analyses revealed that only a small subset of genes was significantly altered, consistent with selective knockdown of miR-21 and its downstream targets, whereas dovitinib alone induced broad transcriptional changes attributable to kinase inhibition and off-target effects. In xenograft mouse models, intraperitoneal administration of **dovitinib-RIBOTAC** maintained in vivo stability, suppressed breast cancer metastasis to the lungs, and significantly reduced metastatic nodules without significant weight loss in mice. Moreover, in a mouse model of Alport syndrome, the compound stabilized albumin levels and improved kidney function, underscoring the therapeutic promise of repurposing protein-targeted drugs as RNA-directed degraders.

To broaden the structural diversity of RNase L recruiters, Meyer et al. reported a new recruiter, derived from 2-benzylidene-3-thiophenone and optimized through DELopen library screening [38]. The new RNase L recruiter was conjugated to dovitinib, yielding **RIBOTAC-7**. This chimera retained comparable binding affinity to pre-miR-21 (K_D_ ≈ 4.6 μM) while taking advantage of a recruiter that offered improved stability and synthetic accessibility. Importantly, the study demonstrated that RNase L recruitment can be achieved by the newly identified small-molecule warheads, not only by canonical 2′-5′A_4_ analogs. Moreover, by pairing this novel recruiter with a ligand of enhanced potency, **RIBOTAC-7** selectively degraded pre-miR-21 in triple-negative breast cancer cells, reducing invasiveness by ~30% at 5 μM, while no effect in normal cells. Nevertheless, despite the incorporation of this novel recruiter, the overall activity of **RIBOTAC-7** remained lower than that of the earlier **C1**-based RIBOTAC. To evaluate the selectivity of the compound, global miRNA profiling of 377 distinct miRNAs was performed, revealing that mature miR-21 was markedly down-regulated together with only five other miRNAs showing more than two-fold changes. These findings suggest that the compound preferentially recognizes structural elements unique to pre-miR-21, thereby inducing selective degradation with minimal off-target effects on other miRNAs.

A subsequent study utilizing RIBOTAC approach was reported for targeting the pri-miR-17-92 cluster, which encodes six different miRNAs. This cluster includes miR-17, miR-18a, and miR-20a, which share similar structural features in their Dicer processing sites. A previous study had developed a dimeric small molecule capable of binding to such Dicer sites [44], resulting in selective suppression of the maturation of miR-17, miR-18a, and miR-20a at the concentrations of 100–500 nM. Utilizing the previous study, the dimeric pri-miR-17-92 cluster ligand was conjugated to **C1**, yielding **7** [39]. This chimera localized in the cytoplasm, where both RNase L and pre-miRNAs are present, and induced selective cleavage of miR-17, miR-18a, and miR-20a. In breast and prostate cancer cell lines, **7** reduced these pri-miR-17-92 cluster RNAs by ~20–36% at 500 nM, while no effect in other cluster members. In addition, global miRNA profiling of 373 miRNAs showed significant alterations in mature miRNAs derived from the 17-92 cluster, with miR-17 and miR-18a being the most prominently affected. This finding indicates that the RIBOTAC strategy is applicable to other microRNAs when suitable selective ligands are identified.

Unlike earlier RIBOTACs that were derived from pre-miRNA processing inhibitors, an important study on miR-155 demonstrated that a fully inactive ligand could be converted into an effective degrader. Using AbsorbArray screening of a natural product-like small-molecule library, a binder targeting the A bulge in pre-miR-155 was identified and optimized. The pre-miR-155 ligand was subsequently conjugated with **C1**, to generate **pre-miR-155 RIBOTAC** [31]. At a concentration of 100 nM, this chimera reduced pre- and mat-miR-155 levels by ~70%. Binding assays with a mutated A-bulge in pre-miR-155 demonstrated that the pre-miR-155 ligand was essential for recognizing the pre-miR-155 bulge. Although several microRNAs, including miR-18a, miR-101-1, miR-1226, miR-3945, and miR-4435, share sequences similar to the pre-miR-155 RIBOTAC binding site, pre-miR-155 possesses a more favorable RNase L cleavage context, resulting in selective degradation of this target. Consistently, global miRNA profiling in MDA-MB-231 cells showed that the pre-miR-155 RIBOTAC selectively reduced miR-155 levels among 373 detected miRNAs, with no detectable off-target effects. Mechanistically, the degrader restored downstream tumor suppressor pathways by ~50% upregulation of SOCS1, a direct target of miR-155. Functional studies further showed that **pre-miR-155 RIBOTAC** suppressed angiogenesis in HUVECs by modulating Von Hippel–Lindau (VHL) protein levels, consistent with the known role of miR-155 [45]. Importantly, in vivo administration in a breast cancer metastasis mouse model reduced lung nodules and inhibited colonization. These findings highlight that even non-functional ligands can be repurposed into potent RIBOTACs when coupled with RNase L recruiter, thereby broadening the scope of degradable RNA targets.

As an alternative strategy of small-molecule RIBOTAC, Fang et al. introduced the Aptamer-RIBOTAC (ARIBOTAC) as a novel approach for tumor-specific degradation of oncogenic miRNAs [40]. Traditional small-molecule RIBOTACs face challenges in identifying selective ligands of precursor miRNAs, while ASO-based RIBOTACs suffer from poor cell permeability and lack of tumor specificity. To overcome these limitations, the authors designed two distinct chimeras: **4A-ASO-AS**, which targets miR-210-3p, and **4A-ASO_155_-AS**, which targets miR-155-5p. In both constructs, an ASO–RNase L recruiter (4A) was conjugated to the aptamer AS1411, which specifically binds nucleolin overexpressed on cancer cells. Thus, each chimera simultaneously directs RNase L recruitment and aptamer-mediated delivery while maintaining target specificity toward different miRNAs. Using miR-210-3p and miR-155-5p as proof-of-concept targets, ARIBOTAC demonstrated efficient and selective miRNA degradation, leading to downstream changes in protein expression, reduced cancer cell migration, and strong antitumor activity in both cell and mouse models. Global miRNA profiling of MCF-7 cells treated with **4A-ASO-AS** revealed that miR-210-3p was the most significantly downregulated miRNA, confirming the selectivity of ARIBOTAC. Notably, ARIBOTAC showed superior efficacy compared to ASO inhibitors, highlighting its potential to enhance therapeutic outcomes.

As another approach, a tumor microenvironment-activated RIBOTAC (TaRiboTAC) was developed to spatially confine RNase L recruitment to the acidic, oxidative conditions characteristic of the tumor microenvironment [41]. TaRiboTAC utilizes bivalent pre-miR-21 binders, a phenylboronic acid-caged RNase L recruiter, a cyclic RGD (cRGD) tumor-homing peptide, and the near-infrared (NIR) fluorophore IR780, yielding **RIBOTAC21-BA**. The amphiphile self-assembles into inert nanoparticles that disassemble at low pH to expose functional modules, while H_2_O_2_-mediated decaging restores recruiter activity, thereby enabling selective degradation of pre-miR-21 in cancer cells with minimal effects in nonmalignant cells. Through cRGD-mediated targeting, **RIBOTAC21-BA** accumulates in tumors, elicits catalase-reversible, H_2_O_2_-dependent pre-miR-21 degradation with downstream PDCD4 restoration, and enhances radiosensitivity. In mice, combination treatment of **RIBOTAC21-BA** and X-ray irradiation induced suppression of A549-Luc tumor growth by ~74% over 18 days without overt systemic toxicity.

### 2.2. RIBOTACs Targeting mRNAs

Many disease-associated proteins, such as transcription factors or intrinsically disordered proteins like α-synuclein, lack defined binding pockets or stable tertiary structures, making it difficult to develop direct protein-targeting drugs. However, their coding mRNAs often contain well-defined, stable secondary or tertiary structural motifs, that can serve as selective and high-affinity binding sites for small molecules. By targeting these structured elements within the mRNA, it becomes possible not only to inhibit translation but also to trigger selective RNA degradation through RIBOTAC (Table 2).

The Disney group, a pioneer in development of RNA-targeted small molecules, reported the first RIBOTAC targeting an mRNA associated with a neurodegenerative disorder. The study focused on repeat expansions of G_4_C_2_ (r(G_4_C_2_)^exp^) in chromosome 9 open reading frame 72 (*C9orf72*) gene, which are causative of amyotrophic lateral sclerosis (ALS) and frontotemporal dementia (FTD). Healthy individuals typically have 2–30 G_4_C_2_ repeats, whereas patients exhibit hundreds [52]. Using the lead identification software, INFORNA 2.0, the authors have previously found a compound, **1**, that binds selectively to 1 × 1 G/G internal loops within the r(G_4_C_2_)^exp^ [53]. To enhance binding affinity, a bivalent version, compound **3**, was synthesized by linking two units of compound **1**. The compound was further connected with the RNase L recruiter generating a RIBOTAC targeting r(G_4_C_2_)^exp^, compound **7** [46]. In c9ALS patient-derived spinal neurons and iPSCs, the r(G_4_C_2_)^exp^-targeting RIBOTAC selectively degraded the mutant *C9orf72* intron-1 transcript up to ~64% reduction at 500 nM, without affecting the healthy allele. Off-target assessment showed that compound 7 selectively cleaved G4C2 repeats longer than 30 units, while *C9orf72* transcripts lacking the repeat expansion (exon 1b variants) remained unaffected. The compound also significantly decreased toxic dipeptide repeat proteins (DPRs) translated from r(G_4_C_2_)^exp^. In a *C9orf72* BAC transgenic mouse model, a single intracerebroventricular injection of 33 nmol RIBOTAC reduced r(G_4_C_2_)^exp^ RNA levels by ~44% and lowered nuclear RNA foci. These results highlight the therapeutic potential for targeting pathogenic RNA in c9ALS/FTD with RIBOTAC technology.

Previous RNA-targeting strategies have primarily focused on rare genetic disorders. To expand the application of RIBOTACs to more common diseases such as cancer, the Disney group reported QSOX1 mRNA targeting RIBOTAC to inhibit the invasion and proliferation of breast cancer cells [47]. The authors performed an in vitro screening using a library of low-molecular-weight compounds bearing diazirine cross-linking modules to get the mRNA binding modules. Upon UV irradiation, the diazirine moiety in the screening compounds generated covalent cross-linking with RNA bases, enabling the identification of binding interactions. Among the screened molecules, compound **F1**, which uniquely lacked aromatic nitrogen atoms, exhibited the highest affinity for QSOX1 mRNA with a K_D_ of 16 µM towards the target QSOX1 mRNA. QSOX1 is a well-characterized oncogene implicated in breast cancer progression and metastasis. **F1** was subsequently converted into a RIBOTAC (**F1-RIBOTAC**), which achieved a 35% reduction in QSOX1-a mRNA levels at 10 µM concentration without affecting the expression of the alternative isoform QSOX1-b. Moreover, **F1-RIBOTAC** treatment resulted in a 40% decrease in the invasiveness of MDA-MB-231 breast cancer cells. This study provides the first evidence that inactive RNA-binding small molecules can be transformed into functional RNA-degrading agents through RIBOTAC design.

With the similar screening approach with diazirine moiety, the Disney group identified a ligand, compound **F3**, which targets another oncogenic protein galectin-1 (LGALS1) mRNA [50]. **F3** was converted into LGALS1 mRNA RIBOTAC by conjugating RNase L recruiter. Notably, **F3-RIBOTAC** selectively targeted the mRNA of LGALS1, reducing LGALS1 mRNA level into ~46% and LGALS1 protein level into ~40% at 10 μM concentration. In addition, **F3-RIBOTAC** attenuates migration and viability in breast cancer cells, with only modest effects on additional low-abundance transcripts (PRNP, SNHG5, and CUTA). Together with the QSOX1 mRNA RIBOTAC, this study highlights that targeted RNA degradation strategies can be expanded beyond rare genetic disorders to more common cancers.

To further investigate the impact of linker design on RIBOTAC performance, three F3-RIBOTAC variants were synthesized with three different polyethylene glycol (PEG) linker lengths. These were tested both in vitro and in MDA-MB-231 cells for LGALS1 mRNA cleavage activity. The shortest linker, **F3-RIBOTAC**, displayed the highest activity (55% in vitro, 38% in cells), whereas longer linkers showed progressively reduced efficacy.

Cellular uptake assays confirmed that extended linkers markedly decreased intracellular accumulation, indicating that steric effects and permeability jointly influence RIBOTAC activity. These results highlight the importance of optimizing linker architecture to balance molecular flexibility, target affinity, and cellular delivery. Transcriptome-wide, unbiased profiling further revealed that, among 111 RNAs bound by F3, only four transcripts (LGALS1, PRNP, SNHG5, and CUTA) were significantly cleaved by **F3-RIBOTAC**. Together with the QSOX1 mRNA RIBOTAC, these studies represent that targeted RNA degradation strategies can be extended beyond rare genetic disorders to more prevalent cancers.

mRNAs often have structured elements within the 5′ untranslated region (5′ UTR), which serves as a critical site for ribosome binding and initiation of translation. With this aspect, the Disney group has developed a strategy to selectively target the internal ribosome entry site (IRES) located in the 5′ UTR to induce mRNA degradation utilizing RIBOTAC technology. The authors successfully achieved selective degradation of the mRNAs encoding well-known oncogenic proteins such as c-Myc and c-Jun [31]. These proteins are representative examples of proteins considered “undruggable” by conventional small-molecule inhibitors. To identify the selective ligand of c-Myc and c-Jun IRES, INFORNA 2.0 software was utilized, resulting in the identification of c-Jun mRNA binder in a 5′UAU/3′A_A bulge and c-Myc mRNA binder in 5′UUCG/3′ACCC internal loop. These identified molecules targeting c-Jun and c-Myc, the c-Jun-binder and c-Myc-binder, showed with K_D_ values of 1.1 µM and 2.3 µM respectively, without any effect on mRNA expression level. Each mRNA binder was converted into **JUN-RIBOTAC** and **c-Myc-RIBOTAC** by conjugating RNase L recruiter. The **JUN-RIBOTAC** effectively reduced JUN-mRNA levels by 40% and suppressed the protein levels by 75% in Mia PaCa-2 cells with a 2µM concentration. It also reduced the invasion by 60% and cell proliferation by 40% in MiaPaCa-2. In addition, **c-Myc-RIBOTAC** reduced MYC-mRNA levels by 50% at a 10 µM, with the suppression of the protein levels by 50% in HeLa, MDA-MB-231, and Namalwa cells. Global RNA profiling revealed that only 84 transcripts (0.40%) were significantly affected, confirming the high selectivity of MYC-RIBOTAC. The most downregulated transcript was EGR1, a well-known downstream target of MYC, providing strong evidence that MYC-RIBOTAC acts through the intended pathway. This study represents a representative example of targeting a long-considered “undruggable” protein at the mRNA level, demonstrating the feasibility of transcript-directed degradation for challenging oncogenic targets.

To broaden the targetability of undruggable proteins, the Disney group explored RIBOTACs targeting intrinsically disordered proteins (IDPs). α-Synuclein is one of the well-known IDPs lacking stable tertiary structure and defined ligand-binding pockets, making it a highly challenging target with conventional small-molecule drug. To address this, Tong et al. developed an RNA-targeted approach by identifying small molecules that bind to a structured iron-responsive element (IRE) in the 5′ UTR of SNCA mRNA, which encodes α-synuclein [49]. Through screening of an RNA-focused chemical library, they identified **Synucleozid-2.0**, a selective binder to the A-bulge of the SNCA IRE, which inhibits ribosomal assembly and reduces α-synuclein translation. Synucleozid-2.0 was then converted into a RIBOTAC, **Syn-RiboTAC**, by tethering it to an RNase L recruiter, enhancing its potency and enabling selective degradation of IRE-containing SNCA transcripts. **Syn-RiboTAC** achieved a ~50% reduction in SNCA mRNA and a >60% reduction in protein levels in SH-SY5Y cells, while sparing structurally unrelated transcripts. In patient-derived dopaminergic neurons, **Syn-RiboTAC** significantly decreased α-synuclein levels and rescued ~50% of aberrantly expressed genes, demonstrating transcriptomic and proteomic selectivity. This study exemplifies how RNA-targeting small molecules and their conversion to RIBOTACs can expand the druggable landscape to previously intractable targets like α-synuclein.

Inspired by the RIBOTAC strategies that utilize RNA-binding small molecules connected to the recruiter of RNase L for catalytic RNA degradation, a recent study introduced an inducible RIBOTAC (**iRIBOTAC**) platform that allows stimulus-dependent activation of RNA cleavage [50]. The **iRIBOTAC** was generated by conjugating a bivalent G-quadruplex (G4) RNA-binding ligand with a caged RNase L recruiter. This recruiter stays inactive until it is specifically triggered by factors such as a phosphine-mediated Staudinger reaction, tumor-associated enzymes (e.g., NQO1), or elevated metabolites (e.g., H_2_O_2_, selenocysteine), allowing precise control of RNase L activation through spatial and temporal selection. Upon activation, **iRIBOTAC**s effectively degrade oncogenic G4 RNAs such as c-Myc, NRAS, KRAS, and ADAM10 at low micromolar concentrations (IC_50_ ≈ 5.2 μM), while showing minimal off-target effects in the absence of activation. After activation of **iRIBOTAC**, transcriptome-wide RNA sequencing identified differentially expressed genes. Among the top 100 downregulated transcripts, 69 contained consensus G-quadruplex motifs, indicating selective degradation of G4-containing RNAs, whereas the remaining genes likely represent off-target or secondary regulatory effects. Importantly, this study established a representative strategy for modulating RIBOTAC function through external stimuli, demonstrating that RNA degradation activity can be conditionally controlled in response to specific cellular signals.

### 2.3. RIBOTACs Targeting Viral RNAs

Not only human RNAs but also viral RNAs have been targeted using RIBOTAC technology. COVID-19, a global pandemic, is caused by the RNA virus SARS-CoV-2. Upon infection, the viral RNA propagates within the human body and replicates its genomic information, leading to severe respiratory symptoms, particularly in the bronchi. One promising therapeutic strategy involves targeting structured RNA elements within the viral genome, most notably the frameshifting element (FSE), which regulates the translation of essential viral polyproteins. The FSE mediates a −1 ribosomal frameshift that enables the translation machinery to shift reading frames and translate ORF1b immediately following ORF1a. This event is critical, as ORF1b encodes key enzymes necessary for viral replication, including helicase, RNA-dependent RNA polymerase, and exonuclease.

To target this structure, Haniff et al. conducted a microarray screen to identify small molecules that bind the SARS-CoV-2 FSE and subsequently converted a lead compound into a RIBOTAC [51] (Table 2). The FSE structure comprises an attenuator hairpin (AH) featuring a 1 × 1 UU internal loop, which was selectively recognized by a small molecule named **C5** with a binding affinity at a concentration of 11 nM. **C5** reduced frameshifting efficiency by 25% at a 2 µM concentration without exhibiting cytotoxicity. However, **C5** alone did not reduce the FSE RNA levels. To enhance efficacy, the authors developed **C5-RIBOTAC** by conjugating the RNase L recruiter to C5, enabling targeted degradation of the FSE RNA. **C5-RIBOTAC** effectively cleaved SARS-CoV-2 RNA and significantly reduced reporter signal at 2 µM, with no observed activity against SARS-CoV RNA. This study exemplifies the potential of RIBOTAC to selectively degrade viral RNAs and presents a novel approach for antiviral drug development.

## 3. Bleomycin Conjugated Degraders

Bleomycins are a group of natural glycopeptides with anti-tumor and antibiotic effects, which were isolated from the fermentation broth of Streptomyces verticillus [54]. Bleomycin cleaves DNA through oxidative damage mediated by its metal-binding domain, which complexes with Fe(II) and activates molecular oxygen. This activated complex generates reactive oxygen species that induce single- and double-strand breaks, primarily at specific DNA sequences rich in GT/GC sites. Such DNA cleavage underlies its potent cytotoxic and antitumor activity. Angelbello et al. exploited the oligonucleotide-cleaving activity of bleomycin to design **Cugamycin** (Table 3), a selective RNA degrader for myotonic dystrophy type 1 (DM1) [33]. DM1 arises from expanded CTG repeats in the 3′ UTR of the DMPK gene, producing r(CUG)^exp^ that sequesters MBNL1 and disrupts splicing. By identifying internal UU loops within r(CUG)^exp^, they developed a dimeric binder conjugated to bleomycin A5, enabling targeted cleavage. Unlike bleomycin, which broadly cleaves DNA and activates DNA damage pathways, **Cugamycin** showed selective activity, cleaving 40% of DMPK transcripts in patient-derived myotubes (DC_50_ ≈ 0.78 µM) without affecting healthy cells or short repeat tracts. In an in vivo mouse model study, intraperitoneal administration achieved efficient muscle distribution and reduced toxic r(CUG)_250_ RNA by ~40% in DM1 mouse models, thereby ameliorating splicing defects. In vehicle-treated DM1 (HSALR) mice, extensive mis-splicing events and 326 dysregulated genes were observed. Treatment with **Cugamycin** restored normal splicing patterns and normalized the expression of nearly half of these dysregulated genes, while leaving unaffected genes unchanged. This study represents the first proof-of-concept that linking a directly cleaving warhead to an RNA-binding ligand can confer selective target degradation.

To further expand the scope of targetable repeat expansion RNAs, Benhamou et al. developed a bleomycin-conjugated degrader targeting r(CCUG)^exp^ in CNBP pre-mRNA, the causative mutation in myotonic dystrophy type 2 (DM2) [59]. r(CCUG)^exp^ sequesters MBNL1, resulting in widespread splicing defects. They optimized a dimeric 6′-acylated kanamycin binder of 2 × 2 internal loops [55], which bound r(CCUG)_12_ with high affinity (K_D_ ≈ 97 nM), and conjugated it to bleomycin A5 to generate **Compound 2**. This chimera selectively cleaved RNA, achieving ~50% cleavage of r(CCUG)_10_ at 2.5 µM and nearly complete cleavage at higher concentrations. In DM2 fibroblasts, **Compound 2** reduced intron 1 abundance by 50% at 5 µM, while the parent binder alone achieved only 20% reduction. Importantly, unlike bleomycin, **Compound 2** did not induce DNA damage signaling and showed no cleavage activity toward nonpathogenic short CCUG repeats in healthy fibroblasts, highlighting its selective and safe RNA-targeted degradation. Together, these results highlight that bleomycin-conjugated degraders can be engineered to discriminate between toxic repeat expansions and normal transcripts, offering a safe and effective strategy for selectively targeting pathogenic RNAs in trinucleotide and tetranucleotide repeat disorders.

Since the binding affinities of earlier ligands were not sufficiently strong, a new scaffold was sought using an alternative screening strategy. Gibaut et al. employed solid-phase DNA-encoded library (DEL) screening to identify a novel small-molecule binder of r(CUG)^exp^ [56], which forms numerous 1 × 1 UU internal loops arranged in a stable hairpin structure. The resulting hit, **DEL1**, was conjugated to bleomycin A5 via its terminal amine to generate **DEL1-Bleo**, which displayed ~6-fold higher binding affinity compared to the parent binder (K_D_ ≈ 1.1 µM). In cleavage assays with radiolabeled r(CUG)_10_, 10 µM of **DEL1-Bleo** achieved ~60% RNA degradation. In patient-derived myotubes, treatment with 5 µM **DEL1-Bleo** reduced DMPK mRNA levels by ~25% without inducing DNA damage, as confirmed by γ-H2AX staining. Furthermore, short nonpathogenic CUG repeats and wild-type myotubes remained unaffected, underscoring the selectivity imparted by structural differences between long and short repeats.

To demonstrate that bleomycin-conjugated RNA degrader can also act beyond mRNA 3′ UTRs and extend to non-coding RNAs, Liu et al. developed a bleomycin-conjugated degrader directed against the oncogenic miRNA-17-92 cluster implicated in multiple cancers and genetic diseases [39]. A previously identified small-molecule binder of Drosha/Dicer processing sites was optimized into a dimer, achieving significantly improved binding affinity (K_D_ ≈ 120 nM). Conjugation of this dimer with bleomycin A5 yielded a chimera, **compound 5**, that selectively cleaved precursor miRNAs. In MDA-MB-231 cells, 100 nM of the **compound 5** reduced pri-miR-17-92 levels by ~33% and decreased pre-miR-17 and pre-miR-20 levels by 33% and 46%, respectively, while mature cluster members were reduced by 25–40% at 500 nM. In DU-145 cells, the chimera showed even greater potency, cleaving pri-miR-17-92 at concentrations as low as 10 nM, with DC_50_ values in the low nanomolar range. These findings provide strong evidence that directly cleaving warheads conjugated to selective RNA binders can effectively target precursor microRNAs, highlighting the versatility of this approach beyond repeat expansions or mRNA 3′ UTRs. In addition, global miRNA profiling of 373 miRNAs demonstrated selective degradation of miR 17-92 cluster, while miR-17 and miR-18a being strongly affected.

With the similar approach, Suresh et al. developed a pre-miR-372 degrader inspired by the **JUN-RIBOTAC**, which targets a 5′UAU/3′A_A bulge in c-Jun-SL-1. While JUN mRNA has three unpaired uridines that favor RNase L cleavage [31], pre-miR-372 contains the same bulge motif near the Dicer processing site. However, it is flanked by three AU base pairs and six purines, creating a sequence context better suited for bleomycin-mediated cleavage [57]. To exploit this, the authors replaced the RNase L recruiter in **JUN-RIBOTAC** with bleomycin while keeping the same RNA-binding moiety, thus retargeting the chimera from JUN-mRNA to pre-miR-372. In vitro FRET assays confirmed selective cleavage of pre-miR-372 by the degrader, **compound 3**, at a concentration of 1.2 μM, without activity on JUN-mRNA or RNase L-dependent cleavage. Transcriptome analysis of approximately 22,000 genes revealed that only 476 (2.1%) were significantly affected, indicating high selectivity of the degrader. Consistently, global miRNA profiling following **compound 3** treatment showed a ~35% reduction in miR-372 levels, while changes in other miRNAs were minimal. In AGS cells, treatment reduced mat-miR-372 levels by 28% and 50% at 0.2 and 2 μM, respectively, with no effect on JUN-mRNA. This derepression of the direct miR-372 target LATS2 increased LATS2 protein abundance and inhibited cell proliferation by 16% and 34% at the same concentrations. In contrast, NCI-N87 cells, which express ~16-fold lower levels of miR-372, showed no significant antiproliferative response, representing the degrader’s dependence on pre-miR-372 abundance.

## 4. Deglyco-Bleomycin Conjugated Degraders

Bleomycin consists of three functional domains: the N-terminus, which determines metal chelation, O_2_ activation, and DNA cleavage selectivity; the C-terminus, which contributes to DNA-binding affinity; and the carbohydrate domain, which accommodates the Fe(II)-O_2_ complex, enhances cell permeability, and can form hydrogen bonds with DNA to promote efficient cleavage [60]. Accordingly, bleomycin-conjugated degraders have been associated with unintended side effects arising from off-target DNA cleavage. Removal of the disaccharide yields deglyco-bleomycin, which displays 2–5-fold reduced DNA cleavage compared to bleomycin, suggesting a potential increase in RNA selectivity. Based on this rationale, Angelbello et al. [58] developed **deglyco-cugamycin** by removing the disaccharide moiety of bleomycin (via HF–pyridine cleavage) while retaining the r(CUG)exp-binding module from the parent **Cugamycin** (Table 3).

Biochemical assays showed that **deglyco-cugamycin** maintained RNA cleavage activity, achieving ~35% cleavage of r(CUG)_10_ at 1 μM, comparable to the parent compound. Importantly, whereas **Cugamycin** induced DNA cleavage at 500 nM in the absence of r(CUG)_12_, **deglyco-cugamycin** showed no DNA cleavage under the same conditions, confirming improved RNA selectivity. Cellular studies further supported this conclusion: treatment with up to 25 μM **deglyco-cugamycin** caused no DNA damage response in DM1 patient-derived myotubes, and live-cell fluorescence microscopy demonstrated similar permeability for both **Cugamycin** and **deglyco-cugamycin**. Functionally, **deglyco-cugamycin** reduced r(CUG)exp–MBNL1 nuclear foci by 40% and rescued MBNL1 exon 5 splicing defects at 2 μM. Taken together, **deglyco-cugamycin** preserved the efficacy of the parent cugamycin while exhibiting enhanced RNA selectivity and reduced DNA-associated toxicity, making it a promising degrader design.

## 5. Imidazole Conjugated Degraders

While RIBOTACs harness the endogenous RNase L protein to achieve selective RNA degradation by proximity recruitment, an alternative strategy that directly mimics the catalytic principles of ribonucleases has been reported. Among ribonucleases, RNase A has a unique ability to cleave an RNA strand by the histidine residue functioning as key catalytic center for phosphodiester bond cleavage. In RNase A, His12 functions as a general base, abstracting a proton from the 2′-hydroxyl group of the RNA backbone to initiate nucleophilic attack and form a cyclic phosphate intermediate. Consequently, His119 donates a proton to the departing 5′-oxygen, facilitating bond scission, while Lys41 and Phe120 stabilize the pentavalent phosphorus transition state to ensure efficient catalysis [61].

This mechanistic insight inspired the design of synthetic small molecules that incorporate histidine or imidazole moieties to reproduce RNase-like reactivity, thereby promoting RNA cleavage independent of cellular nucleases (Table 4). A representative example was reported by Martin and colleagues, who coupled the aminoglycoside antibiotic neomycin, a known RNA binder, with histidine or imidazole to generate RNA degraders targeting the HIV-1 TAR RNA [62]. The resulting conjugates, **compound 3** (histidine degrader) and **compound 4** (imidazole degrader), exhibited enhanced binding and functional activity compared with neomycin alone. Notably, **compound 3** exhibited clear site-specific cleavage by interacting with the bulge region of TAR RNA in a concentration of 5 μM. These results provided proof of concept that histidine- and imidazole-linked small molecules can mimic RNase A catalysis and induce site-specific RNA cleavage [63].

Building on this principle, Mikutis et al. introduced Proximity-Induced Nucleic Acid Degraders (PINADs) by linking RNA-binding ligands to an imidazole warhead [34]. Two representative PINADs were developed to target distinct structured elements within the SARS-CoV-2 genomic RNA: **PDS-imi6**, which cleaves G-quadruplex (G4) regions, and **MTDB-imi6**, which cleaves the β-coronaviral pseudoknot required for viral frameshifting. In VERO-CCL-81 cells infected with SARS-CoV-2, **PDS-imi6** suppressed viral replication with an IC_50_ of 1 μM, without affecting host cell viability. Degradation of G4s was observed only under potassium-rich conditions that stabilize G-quadruplex structures, whereas no cleavage occurred in lithium-containing buffers that destabilize them, confirming structural selectivity of the compound. Similarly, **MTDB-imi6** demonstrated potent activity in vitro and in cells. **MTDB-imi6** effectively degraded viral RNAs in a concentration of 2–6 μM. No degradation of pseudoknot of SARS-CoV-2 is observed with mutated pseudoknot constructs, confirming high structural specificity. In mouse models, intranasal administration of **MTDB-imi6** both before and shortly after SARS-CoV-2 infection significantly reduced viral burden in the lungs. Collectively, these studies demonstrate that imidazole-conjugated RNA binders can reproduce RNase-like catalytic activity, positioning PINADs as a versatile and nuclease-independent approach for targeted RNA degradation.

## 6. Discussion

### 6.1. Comparative Analysis for Small-Molecule RNA Degraders

Among small-molecule RNA degraders, each modality presents distinct advantages and limitations (Table 5). RIBOTACs achieve high selectivity and catalytic turnover by recruiting endogenous RNase L to the target RNA, enabling enzyme-mediated degradation. However, their dependence on RNase L expression levels and relatively large molecular size may result in variable pharmacokinetic performance across cell types and restrict intracellular delivery. Despite these limitations, RIBOTACs represent the most advanced platform, with multiple preclinical studies demonstrating reproducible RNA knockdown in cancer and neurodegenerative disease models, although none have yet progressed to clinical evaluation.

Bleomycin-conjugated degraders exhibit potent oxidative RNA-cleaving activity once localized to the target, providing strong degradation efficiency. Nevertheless, their therapeutic applicability remains limited due to low selectivity, requirement for metal ions and oxygen for activation, and potential genotoxicity. These systems are currently classified as early preclinical, having been validated primarily in biochemical and cellular assays with limited in vivo data.

In contrast, imidazole-based degraders represent a chemically minimalist and fully synthetic approach. Their relatively low molecular weight enhances cellular permeability, while their design allows tunable reactivity and improved physicochemical stability. However, their catalytic efficiency and selectivity remain modest compared with enzyme-recruiting systems. To date, imidazole-based degraders have demonstrated robust RNA cleavage both in vitro and in cells, remaining distant from clinical translation but emerging as promising early preclinical candidates.

Among current RNA degrader platforms, RIBOTACs have reached the preclinical stage, supported by consistent in-cell and in vivo efficacy across multiple disease models, along with preliminary pharmacokinetic and safety evaluations. In contrast, bleomycin- and imidazole-based degraders remain at an earlier preclinical stage, showing in-cell and limited in vivo proof-of-concept activity without comprehensive PK or toxicity assessment. Collectively, these modalities occupy different positions along the translational spectrum, with RIBOTACs leading as a validated enzymatic platform and bleomycin- and imidazole-based degraders emerging as promising chemical alternatives under early exploration.

Taken together, these RNA degrader modalities exhibit distinct yet complementary functional characteristics. Because each degrader displays a unique cleavage preference—with RIBOTACs favoring AU-rich single-stranded regions recognized by RNase L, bleomycin conjugates cleaving near guanine-rich purine sites, and imidazole-based degraders acting efficiently at accessible A/U-rich loops—the choice of modality should be guided by the sequence and structural features of the target RNA as well as cellular RNase L expression levels. Accordingly, these systems can be selectively applied in a context-dependent and mutually complementary manner, enabling flexible and effective RNA degradation across diverse biological systems.

### 6.2. Current Unmet Needs for RIBOTAC

Although RIBOTACs currently represent the most clinically advanced platform among small-molecule RNA degraders, their translation into therapeutic applications still faces several key challenges.

First, **C1**, the RNase L recruiter most commonly used to date, has sub-micromolar cellular efficacy and often requires comparatively high concentrations to achieve sub-stoichiometric cleavage. This result indicates a need for next-generation recruiters with improved potency, metabolic stability, and cellular pharmacology with further structure-activity relationship (SAR) studies. In parallel, quantitative analysis is needed to measure ternary complex cooperativity beyond binary complex formation with RNA and ligand. Methods that enable precise evaluation of RNA-ligand interaction, RNase L activation, and productive proximity will be essential to convert proof-of-principle activity into drug-like efficiency.

A second challenge to overcome is the diversification of RNA decay enzymes. Previous studies have demonstrated that RIBOTACs show no activity in cell lines with low RNase L expression, highlighting the enzyme’s critical role in determining cellular efficacy [31]. This dependency limits the applicability of RIBOTACs to certain cell types with sufficient RNase L abundance. To broaden their utility, it will be necessary to explore alternative RNA-cleaving enzymes and diversify the recruited nucleases. Expanding the enzymatic repertoire to include other RNA endonucleases and exonucleases is expected to facilitate efficient RNA degradation across different cellular environments while maintaining selectivity, ultimately broadening the therapeutic scope of RIBOTACs.

Third, RIBOTACs share conceptual similarities with proteolysis-targeting chimeras (PROTACs) as bifunctional molecules linked by a spacer that induces target degradation. In PROTACs, linker design has been extensively optimized and shown to be a critical determinant of efficacy. Despite these parallels, the linker chemistry of RIBOTACs remains largely unexplored. Except for a few reports [64], systematic investigations are lacking regarding how linker length, rigidity, and physicochemical properties influence ternary complex cooperativity, selectivity, intracellular delivery, and endosomal escape. Accordingly, comprehensive linker SAR studies are required to establish predictive design principles that integrate ternary complex geometry with drug-like properties.

Finally, attention should be given to developability factors that influence pharmacological performance and clinical translation. Many RIBOTACs fall within the 800–1200 Da molecular weight range, which can limit cell permeability, tissue distribution, and susceptibility to efflux. Furthermore, as observed in PROTACs, the so-called hook effect may occur at high concentrations, where nonproductive binary complexes are favored over productive ternary assemblies [65]. In addition, sustained RNA depletion can provoke compensatory transcriptional responses that restore cellular homeostasis. For instance, feedback upregulation of the same transcript, induction of functionally redundant RNAs, or activation of alternative signaling pathways may attenuate long-term knockdown efficacy. Moreover, variability in RNase L expression or activity among different cell types could influence both durability and resistance profiles. While experimental evidence for these limitations remains limited, PK-guided optimization and transcriptome-wide profiling will be invaluable for the rational design of next-generation RIBOTACs with improved pharmacokinetic properties and translational potential.

In summary, the development of more potent recruiters, diversification of degradation mechanisms, systematic linker optimization, and proactive consideration of drug-like properties will be crucial to transform RIBOTACs from compelling proof-of-concept molecules into a robust and clinically viable therapeutic platform.

## 7. Conclusions and Future Perspective

In conclusion, small-molecule-induced RNA degradation has emerged as a compelling therapeutic strategy. Although RNAs were long regarded as difficult to drug due to their structural heterogeneity and lack of canonical binding pockets, recent studies have shown that selective ligands, when conjugated to recruiter modules or direct-cleaving warheads, can promote efficient and catalytic RNA degradation with high specificity.

Nevertheless, further optimization is required before this approach can be translated into clinically applicable modalities. Advancing the field will depend on the development of next-generation recruiters with improved potency and pharmacological properties, expansion of degradation mechanisms beyond RNase L, and systematic investigations of linker chemistry. In addition, addressing pharmacokinetic liabilities and establishing standardized methods to profile on- and off-target effects will be critical for clinical translation.

Small-molecule RNA degraders represent a promising therapeutic modality that broadens the druggable space to encompass both coding and noncoding RNAs, offering new opportunities for treating diseases that remain inaccessible to conventional protein-targeted approaches.

## Figures and Tables

**Table 1:** Reported RIBOTACs targeting miRNAs.

RNA Degrader	Target RNA	Structure	DC_50_ ^a^	In Vivo	Ref.
**RIBOTAC-2**	Pri-miR-96	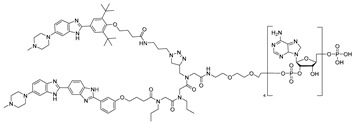	20 nM	200 nM	[36]
**miR-21-RIBOTAC**	Pre-miR-21	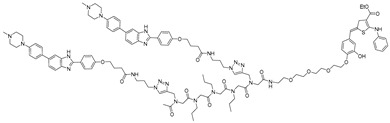	~50 nM	10 mg/kg	[28]
**Dovitinib-RIBOTAC**	Pre-miR-21	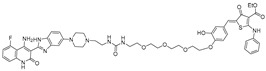	~5 µM	56 mg/kg	[37]
**RIBOTAC 7**	Pre-miR-21	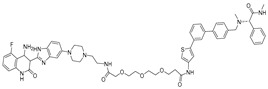	~5 μM	-	[38]
**7**	miR-17-92 cluster	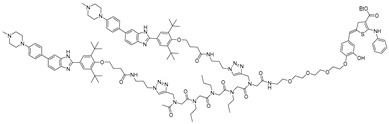	~500 nM	-	[39]
**pre-miR-155 RIBOTAC**	Pre-miR-155	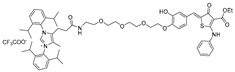	100 nM	1 mg/kg	[31]
**4A-ASO-AS**	miR-210-3p	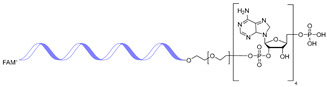	100 nM	-	[40]
**4A-ASO155-AS**	miR-155-5p	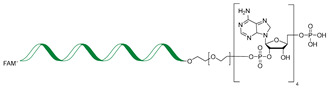	50 nM	5 mg/kg	[40]
**RIBOTAC21-BA**	Pre-miR-21	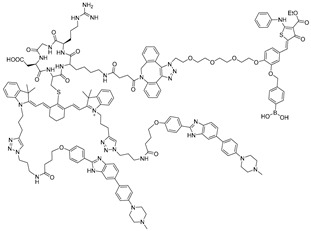	~50 nM	-	[41]

^a^ Half-maximal degradation concentration.

**Table 2:** Reported RIBOTACs targeting mRNAs and viral RNAs.

RNA Degrader	Target RNA	Structure	DC_50_^a^	In Vivo	Ref.
**r(G_4_C_2_)^exp^-targeting RIBOTAC (7)**	*C9orf72* HRE	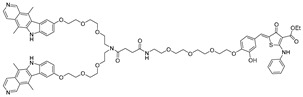	500 nM	33 nmol	[46]
**F1-RIBOTAC**	QSOX1-a mRNA	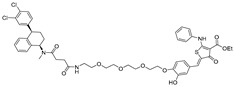	20 µM	-	[47]
**F3-RIBOTAC**	LGALS1 mRNA	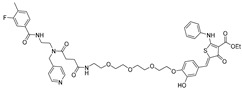	10 µM	-	[48]
**JUN-RIBOTAC**	JUN mRNA	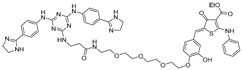	2 µM	-	[31]
**c-MYC-RIBOTAC**	c-MYC mRNA	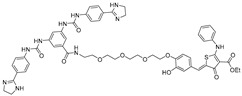	10 µM	-	[31]
**Syn-RIBOTAC**	SNCA mRNA	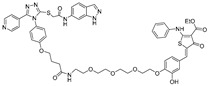	2 µM	-	[49]
**iRIBOTAC**	G-Quadruplex	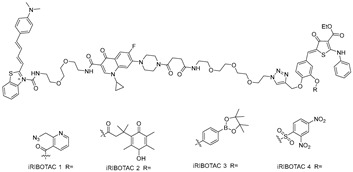	5.2 µM	100 µM (100 µL)	[50]
**C5-RIBOTAC** ^b^	SARS-CoV-2-FSE	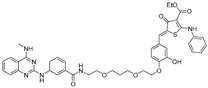	~8 µM	-	[51]

^a^ Half-maximal degradation concentration. ^b^ Reported viral RNA targeting RIBOTAC.

**Table 3:** Reported Bleomycin conjugated and deglyco-bleomycin conjugated RNA degraders.

RNA Degrader	Target RNA	Structure	DC_50_^a^	In Vivo	Ref.
**Cugamycin**	r(CUG)^exp^	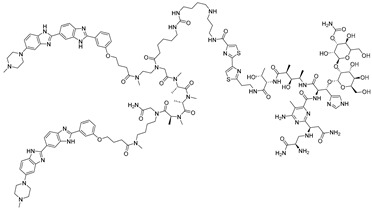	2 µM	10 mg/kg	[33]
**Compound 2**	r(CCUG)^exp^	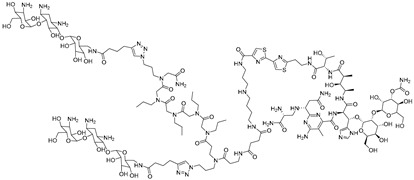	~5 μM	-	[55]
**DEL1-Bleo**	r(CUG)^exp^	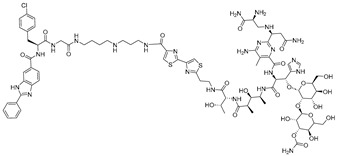	>10 μM	-	[56]
**Compound 5**	miR 17-92 Cluster	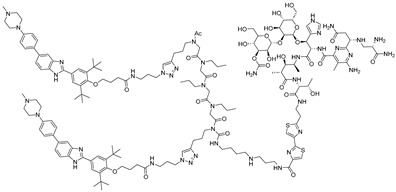	~500 nM	-	[39]
**Compound 3**	Pre-miR-372	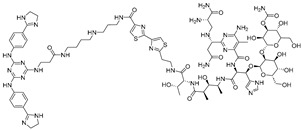	>2 μM	-	[57]
**Deglyco-Cugamycin** ^b^	r(CUG)exp	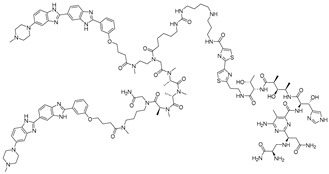	>1 µM	-	[58]

^a^ Half-maximal degradation concentration. ^b^ Reported deglyco-bleomycin conjugated RNA degrader.

**Table 4:** Reported imidazole conjugated RNA degraders.

RNA Degrader	Target RNA	Structure	DC_50_ ^a^	In Vivo	Ref.
**Compound 3,4**	HIV-1 TAR RNA	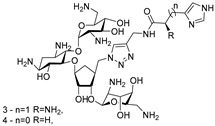	~0.5 μM	-	[62]
**PDS-imi6**	G-quadruplex of SARS-Cov-2 RNA	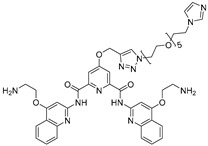	0.75 µM	-	[34]
**MTDB-imi6**	Pseudoknot of SARS-CoV-2 RNA	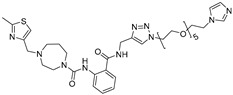	2 µM	25 mg/kg	[34]

^a^ Half-maximal degradation concentration.

**Table 5:** Comparison of RNA degraders.

Modality	Mechanism	Preferred Cleavage Site	Recruiter	Pros	Cons	Stage
RIBOTAC	Enzymatic cleavage by RNase L recruitment	UN^N AU-rich single-stranded loops	2′ 5′ A4 C1 C2	High selectivity High catalytic activity	Depends on the RNase L expression	Pre-clinical
Bleomycin-conjugated degrader	Direct scission by oxidative cleavage	Purine-rich Loop regions	Bleomycin Deglyco-bleomycin	Potent cleavage	Off-target effect Genotoxic risk	Early pre-clinical
Imidazole-conjugated degrader	Direct phospho diester Backbone cleavage	A/U-rich Single-stranded	Imidazole	Lower molecular weight Applicable in all cells	Low selectivity	Early pre-clinical

## Data Availability

No new data were created or analyzed in this study. Data sharing is not applicable to this article.

## Abbreviations

The following abbreviations are used in this manuscript:

2DCS	Two-Dimensional Combinatorial Screening
ADAM10	A Disintegrin and Metalloproteinase Domain-Containing Protein-10
AH	Attenuator Hairpin
ALS	Amylotrophic Lateral Sclerosis
ARIBOTAC	Aptamer-Ribonuclease Targeting Chimera
ASO	Anti-sense Oligonucleotide
CNBP	CCHC-type zinc finger nucleic acid binding protein
cRGD	cyclic Arginyl-glycyl-aspartic acid
CUTA	CutA divalent cation tolerance homolog protein
DEL	DNA-Encoded Library
DM1	Myotonic Dystrophy type 1
DM2	Myotonic Dystrophy type 2
DMPK	Dystrophia Myotonica Protein Kinase gene
DPR	Dipeptide Repeat protein
FOXO1	Forkhead box protein O1
FSE	Frameshifting Element
FTD	Frontotemporal Dementia
HOTAIR	HOX Transcript Antisense Intergenic RNA
HUVEC	Human Umbilical Vein Endothelial Cells
IDP	Intrinsically Disordered Protein
INFORNA	Info-RNA
iPSC	Induced pluripotent stem cell
IRE	Iron Responsive Element
IRES	Internal Ribosomal Entry Site
iRIBOTAC	inducible Ribonuclease Targeting Chimera
KRAS	Kirsten Rat Sarcoma viral oncogene homolog
LGALS1	Lectin, Galactoside-Binding, Soluble, 1 (Galectin-1)
MALAT1	Metastasis-Associated Lung Adenocarcinoma Transcript 1
MBNL1	Muscleblind-Like Splicing Regulator 1
NEAT1	Nuclear Paraspeckle Assembly Transcript 1
NQO1	NAD(P)H:quinone oxidoreductase 1
NRAS	Neuroblastoma RAS viral (v-ras) oncogene homolog
ORF1b	Open Reading Frame-1b
PDCD4	Programmed Cell Death 4
PINAD	Proximity-Induced Nucleic Acid Degrader
PNA	Peptide Nucleic Acid
PRNP	Prion Protein
PROTAC	Proteolysis-Targeting Chimera
QSOX1	Quiescin Sulfhydryl Oxidase 1
QSOX1-b	Quiescin/sulfhydryl oxidase 1b
RIBOTAC	Ribonuclease Targeting Chimera
RISC	RNA-induced Silencing Complex
RTK	Receptor Protein Kinase
SARS-CoV-2	Severe Acute Respiratory Syndrome Coronavirus 2
SNCA	alpha-synuclein
SNHG5	Small Nucleolar RNA Host Gene 5
SOCS1	Suppressor of Cytokine Signaling 1
TAR	Trans-Activation Response element
TaRiboTAC	Tumor microenvironment-activated Ribonuclease Targeting Chimera
Tat	Trans-Activator of Transcription
UTR	Untranslated Region
VHL	Von Hippel–Lindau
XIST	X-inactive specific transcript
